# Need and inequality in the use of health care services in a fragmented and decentralized health system: evidence for Argentina

**DOI:** 10.1186/s12939-020-01168-6

**Published:** 2020-07-31

**Authors:** Alfredo Palacios, Natalia Espinola, Carlos Rojas-Roque

**Affiliations:** Institute for Clinical Effectiveness and Health Policy (IECS), Doctor Emilio Ravignani 2024, Buenos Aires, Argentina

**Keywords:** Inequality, Inequity, Health care disparities, Utilization of health care, Argentina

## Abstract

**Background:**

The high fragmentation and decentralization in the provision of health care services that characterizes Argentina’s health system, as well as the economic and social inequalities, challenge the achievement of the Universal Health Coverage (UHC). The objective of this study is to measure socioeconomic-related inequality and horizontal inequity in the use of health care services in Argentina as well as identify the factors that contribute to these disparities.

**Methods:**

The 2013 National Risk Factor Survey, developed by the Ministry of Health of Argentina, was used to measure socioeconomic-related inequality and inequity in the use of health care services through concentration curves, the Erreygers concentration index, and the index of horizontal inequity. Econometric micro-decomposition was applied to estimate the contribution of each determining factor to inequality in the use of health care services.

**Results:**

The Erreygers concentration index for the use of health care services was 0.1223, evidencing pro-rich inequalities. By adding variables of health care needs, the horizontal inequity index was 0.1296. Non-need factors such as education and health coverage with social security increase pro-rich inequality.

**Conclusions:**

The Argentine health system shows pro-rich inequality in the use of health care services. It is necessary to design strategies to improve articulation between the three coverage subsectors and national, provincial, and municipal governments to keep the commitment of “not leaving anyone behind.” The results showed here could provide lessons for countries with similar contexts and challenges in public health.

## Background

Equity constitutes a social value and a guiding principle of political health action [1]. The Alma-Ata Declaration of 1978, the Ottawa Charter of 1986, and, more recently, the 2030 Agenda for the achievement of the Sustainable Development Goals (SDGs) are a call for social equity and reflect the commitment to design social and health policies with an equity approach. Expressly, objective 10 and goal 3.8 of objective 3 of the SDGs point to the reduction of inequalities in all sectors and achieve Universal Health Coverage (UHC) [2]. Thus, equity as a social value has become increasingly important in international policy agendas.

Since the early 1990s, several Latin American countries have initiated reforms intending to strengthen health systems, reduce inequalities in access to health care services, and expanding the UHC [3]. However, given the gap between the medically possible and financially feasible, some type of explicit or implicit rationing has been inevitable, and on other occasions, the resources are not properly allocated. Consequently, the expansion of coverage and the reduction of health inequalities remains a pending issue in Latin American countries [4].

Argentina is an upper-middle-income country located in South America with a population of 44 million, where 92% live in cities. Noncommunicable diseases account for more than 78% of the burden of disease, and it is one of the leaders in the Latin American region concerning health care expenditure per capita [5]. Compared with other countries in the region, its health care system performs well on several key indicators (for further details, see Table S1 in the Supplementary material). However, there are still some challenges on the public agenda related to both equity and efficiency, as in many other countries in Latin America [3, 6].

In the decade of the 1990s, Argentina underwent a profound reform of its health care system, as well as other countries in Latin America. The purpose was to establish a mechanism that ensured an efficient allocation of resources and guaranteed a more comprehensive provision of health care services based on equity and population needs. During this period, Argentina adopted an ambitious range of reforms, mainly focusing on decentralization and restructuring of social security systems [7].

Currently, the Argentine health system is characterized by decentralization in the public health sector and fragmentation in its social insurance mechanisms, both in the sources of funds and in the structure of service provision [8]. Decentralized functions from the nation to the provinces (and in some cases from the provinces to the municipalities) include fundraising, resource management, setting health goals, setting health strategies and priorities. Health service coverage is fragmented into three subsectors: the public subsector (national, provincial, and municipal), the social insurance subsector (*Obras Sociales*), and the private health subsector. Fragmentation occurs because there are no coordination and cooperation mechanisms in terms of management, financial, and health risks among the three subsectors.

**Fig. 1:**
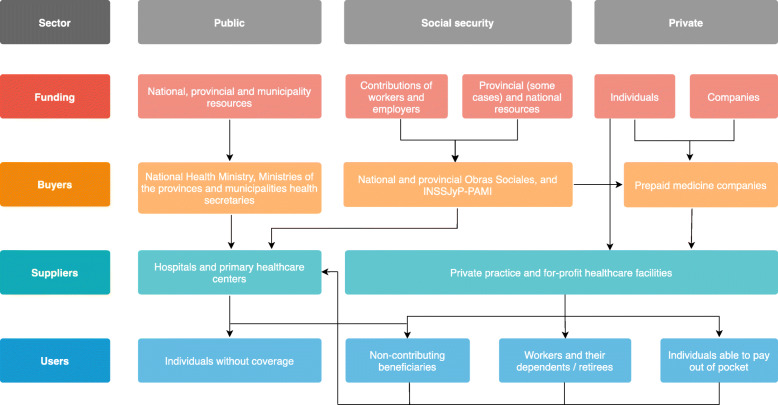
Argentina health system schematic. Source: adapted from Belló et al. 2011 [9]. Notes: INSSJyP: National Institute of Social Services for Retirees and Pensioners

Figure 1 presents the sources from which funds are collected, the organization of insurance structures and risk-sharing schemes, the management mechanisms of the funds, and how these funds translate into the provision of health care services, for each of the three subsectors mentioned [9–11].

The public health subsector is financed by national, provincial, and municipal funds, which contribute to the Ministries or Secretaries of Health at each of these levels. Within this model, there is a notable decentralization in resource management, fundraising, and the establishment of health strategies and priorities. Thus, each province has the autonomy to define and establish a health care strategy for its inhabitants. In terms of provision, the public subsector has a network of public hospitals and primary health care centers that are owned and managed by provincial and municipal authorities. These hospitals and primary health care centers provide free care to anyone who demands it, in general, people without health insurance [6, 9, 12].

Social security, the most important subsector within the health system, is organized around three large groups: i) 269 *Obras Sociales Nacionales* (OSNs); ii) 24 *Obras Sociales Provinciales* (OSPs); and iii) the National Institute of Social Services for Retirees and Pensioners (INSSJyP – PAMI, acronym in Spanish) [9, 11]. OSNs cover over 15 million Argentine salaried workers and their families according to economic activity, whereas OSPs are in charge of providing health care services to public employees in their jurisdiction. The INSSJyP, alternatively, provides coverage to more than 5 million retirees of the national pension system and their families. Currently, 70% of the 269 OSNs have less than 30,000 beneficiaries, and 80% have less than 100,000, which makes them inefficient due to their high administrative costs; in addition, their risk pools are highly unstable to deal with high-cost events [7].

The private health subsector has as a financing mechanism the out-of-pocket payment, destined to the co-payment of services provided under some provincial and national *Obras Sociales*, and the fees and complementary payments of the prepaid insurance provided by prepaid medicine companies, concentrated in four firms with high market power [9–11].

According to the 2010 Population Census, 36.1% of the population does not have formal health coverage; 46.4% had coverage of *Obras Sociales*; private insurance that was accessed through contributions to *Obras Sociales* covered an additional 10.6%; 5.1% accessed prepaid medicine through voluntary insurance payment; only 1.8% were beneficiaries of specific state programs and plans [13]. In terms of total health expenditure, 29% corresponds to public expenditure, 41% corresponds to social security expenditure, and the remaining 30% is private expenditure (which includes out-of-pocket expenses in prepaid insurance, co-payments, and uncovered care). Within public sector expenditure, 67% corresponds to provincial spending [14].

In general, the Argentine health system represents an uncoordinated model for the management of plans and funds, where the contributors with the greatest financial capacity do not direct their contributions towards the subsector that absorbs the most significant financial and health risk, being captured by those subsectors with less financial exposure [7, 11]. This phenomenon of “skimming” between financial coverage systems is complemented by the presence of unwanted cross-subsidies from the public subsector to the rest of the subsectors [15]. That is, the public subsector also provides care to people who have formal insurance, and given their difficulties in billing for the services provided, these practices are challenging to collect and reinvest. All of these aspects reduce the possibilities of individuals without coverage to obtain health care, with implications for the equity of the system.

In particular, the decentralization of the Argentine health system, as well as the absence of mechanisms for redistribution of resources between jurisdiction, leads to significant disparities in terms of public health care expenditure [16], the availability of medical doctors and nurses [17] and infant and maternal mortality rates [18] at the provincial level. By case, the mortality gap in colon-rectum and cervical cancer is 7 and 5.4 times, respectively, between the wealthiest and most impoverished provinces in the country [19]. In the local literature, these cancer mortality gaps have been associated with different provincial capacities for early detection, diagnosis, and treatment of patients [20, 21].

Numerous studies have well documented socio-economic inequalities in Argentina [22–26]. This literature suggests that while socioeconomic inequality is considerable (Gini index was 0.412 in Argentina in 2017) [5], it has declined in recent decades thanks to the implementation of cash transfer programs and the growth of labor formality. While inequalities in access to health care services have been widely described at the local level [27–37], few studies have analyzed the socioeconomic-related inequality in health care variables through the methodological approach presented in this work [38–40].

Nowadays, the Argentine health system is in search of providing effective universal health coverage, meaning that people actually receive prioritized health care services. Among the specific objectives set to achieve this goal, the reduction of disparities in access to health services is one of them [7]. However, this constitutes a significant challenge due to system fragmentation and decentralization - as well as the absence of subsystems integration – leading to inefficiencies and inequities in health [6]. In this context, the objective of this study is to measure socioeconomic-related inequality and horizontal inequity in the use of health care services in Argentina as well as identify the factors that contribute to explain these disparities.

## Methods

### Study design and data

This analytical study used data from the Third National Survey of Risk Factors 2013 (ENFR 2013) designed and compiled by the National Institute of Statistics and Census (INDEC) and the Ministry of Health of Argentina. The ENFR collects information on risk factors, health care utilization, and prevalence of central non-communicable diseases, among others. The sample design of the ENFR is probabilistic, stratified, and multi-stage and is representative at the national and provincial levels with 5000 or more inhabitants. This survey included 32,365 people 18 years of age or older, living in particular households in Argentina. The methodological aspects of the survey to consider for reading the results can be found in the final results report [41].

### Dependent variable

The study-dependent variable is a dichotomous variable that measures the use of health care services (yes / no). The variable is constructed based on the questions referring to whether the individual in the last 30 days consulted a healthcare professional, including a physician (clinician or specialist), dentist, psychologist, psychoanalyst or psychiatrist, or others. The use of health care services meant the respondent reported having consulted at least one of the previously mentioned services.

### Independent variables

The independent variables of the study were selected based on Andersen’s theoretical model of behavior on the use of health care services [42, 43], consistent with other studies that analyze the use of health care services at a regional and local level [44–46]. This model indicates that the use of health care services is a function of three major factors: predisposing factors, enabling factors, and need factors. The predisposing factors are composed of socio-demographic characteristics, characteristics of the social structure, and beliefs in people’s health. The enabling factors correspond to the means and capacity of people to use health care services both at the individual and community levels. The need factors correspond to variables that predispose the use of health care services due to the health problems of the individual.

The study included the following variables as predisposing factors: sex (male / female), age in ranges (18–24 / 25–34 / 35–49 / 50–64 / 65 or more), married or united (yes / no), educational level in ranges (up to incomplete primary / complete primary and incomplete secondary / complete secondary or more) and household size in ranges (1–3 members / 4–6 members / 7 members or more). According to the ENFR 2013, all variables were measured by self-report.

The following variables were included for the enabling factors: type of health coverage (social security insurance / private insurance / public insurance), currently employed (yes / no), income per capita quintiles (I (poorest quintile), II, III, IV, V (richest quintile)), urban populations (between 5000 and 100,000 inhabitants / between 100,001 and 500,000 inhabitants / between 500,001 and 1.5 million inhabitants / more than 1.5 million inhabitants) and geographical region (Great Buenos Aires / Pampeana / Northwest / Northeast / Cuyo / Patagonica). The individual was considered as currently employed if they worked at least 1 h in economic activities during the reference week. Household income includes income from work, retirement, unemployment insurance, scholarships, and other sources of income during the month prior to the survey. In order to estimate the missing values in the income variables, the hierarchical hot-deck approach was used as an imputation method [47]. Because ENFR 2013 does not report information required to estimate the income per capita adjusted by equivalent adult (sex and age of household members), income quintiles were constructed using the income per capita (dividing the total household income by the number of household members).

The grouping of jurisdictions used is in line with the framework of the National Statistical System, that categorizes the country into six statistical regions [47]. The following variables correspond to need factors: self-perceived health status (excellent / very good / good / regular / bad), problems with mobilization (yes / no), feeling lonely or depressed (yes / no), level of physical activity (intense / moderate / low), pain or physical discomfort (yes / no), presence of high blood pressure (yes / no), presence of diabetes mellitus (yes / no), presence of chronic obstructive pulmonary disease (yes / no), presence of chronic kidney disease (yes / no) and cumulative number of chronic diseases suffered (0/1/2/3 or more). The level of physical activity was categorized according to the recommendations of the International Physical Activity Questionnaire (IPAQ). The cut-off points for the different categories are detailed on the IPAQ website or in the ENFR use document [47, 48].

### Statistical analysis

The processing and statistical analysis of the ENFR 2013 database was performed using the Stata® v14.2 statistical software (Stata Corporation, College Station, Texas, USA). The *svy* command was used to specify the weighting factors of the ENFR 2013. For all analyses, statistical significance was considered if *p* < 0.05.

Absolute frequencies and weighted proportions described the socio-demographic characteristics of the population. Absolute frequency, weighted proportions, and the 95% confidence interval described the use of health care services. The concentration curve (CC), the Erreygers concentration index (ECI) and the horizontal inequity index (HI) were computed to measure inequality in the use of health services.

The concentration curve (CC) describes the relationship between the cumulative percentage of the population, ordered by their per capita income, and the cumulative percentage of the use of health services with the diagonal line of equality. Inequality is estimated according to the concavity or convexity of the curve. The further the CC moves away from the line of equality, the greater the degree of inequality. If the CC is below the equality line, there is greater use of health services for the population with higher levels of per capita income. When the CC is above the equality line, it indicates a more significant use by the part of the population with lower per capita income [49].

Nevertheless, a CC does not give a measure of the magnitude of inequality that can be compared across periods, or other relevant variables. For this, the Concentration Index (CI), which is directly related to the CC, does quantify the degree of socioeconomic-related inequality in a health variable [50]. The CI is defined as twice the area between the concentration curve and the line of equality (the 45-degree line). Therefore, when there is no socioeconomic-related inequality, the CI is zero. If the CC lies above the line of equality, the CI takes a negative value, indicating disproportionate concentration of the health variable among poor people; if the CC lies below of the line of inequality, the CI takes a positive value, and it means that there is a disproportionate concentration of the health variable among rich people. In this case, a positive value of the CI means that the use of health care services is higher among the rich. Given the dichotomous characteristic of the dependent variable, the ECI was computed for the methodological advantages in relation to the standard concentration index [51]. However, the interpretation of the index is the same. Mathematically, ECI is obtained:

$$ ECI(y)=\frac{1}{n}{\sum}_{i=1}^n\left[\frac{4{a}_i}{\left({a}^{max}-{a}^{min}\right)}\left(2{R}_i-1\right)\right] $$

In which *a*_*i*_*ϵ*[*a*^*min*^, *a*^*max*^] denotes the dichotomous variable with the limit values 0 and 1, and *R*_*i*_ − 1 denotes the fractional range of per capita income. For any ECI, the values it takes range from −1 to 1, which reflects the variability and strength of the relationship between the variables studied. The values are positive (negative) when there is greater use of health care services for the population with higher (lower) levels of per capita income.

Since variations in the use of health care services due to differences in health status are unavoidable (healthy people use health services less in comparison to non-healthy people), income-related inequality itself is not considered inequity in health care services use. The HI compares the actual distribution of the use of health care services with the expected use according to the health needs of individuals to assess inequities. Therefore, HI measures the degree to which health care use is related to income after controlling for differences in need across the income distribution.

For the estimation of the HI, the use of health care services was standardized following the approach of indirect standardization with non-linear models proposed by O’Donnell [49]. When the health variable is dichotomous, as in our case, this approach suggests using probit or logit models for the standardization of the use of health care services, because it best fits the non-linearity of the distribution of the variable.

There were two stages for the estimation of the HI. First, the use of health care services was estimated using a non-linear model through probit estimation, taking as independent variables a vector of need variables and a vector of no need variables as follows:

$$ {y}_i=\alpha +\beta ln\left({inc}_{\mathrm{i}}\right)+{\sum}_k{\delta}_k{X}_{k1}+{\sum}_p{\varphi}_p{Z}_{p1}+{\mu}_i $$

In which *y*_*i*_ is the observed use of the health care services of an individual *i*, *inc*_*i*_ is the per capita income of the individual *i*, *X* it is a vector of need variables for the use of health care services, *Z*_*p*1_ is a vector of control variables of no need for the use of health care services (predisposing and enabling factors), *α*, *β*, *δ*_*k*_ y *φ*_*p*_ are model parameters and *μ*_*i*_ is the error term. This model allows predicting the probability of using health care services by individuals, i.e., the probability of using health care services that the individual should consume considering that he or she is treated the same to other people that have the same health care needs.

Second, the standardized demand for the use of health care services $\left({\hat{y}}_i^{1s}\right)$ was estimated using the values of *y* predicted by standardizing the *X* variables (health care necessity factors) while simultaneously controlling the *Z* variables (no need factors: predisposing and enabling factors) and the per capita income variable, which arise from the previous regression. Mathematically,

$$ {\hat{y}}_i^x=\hat{\alpha}+\hat{\beta}\ln \left(\overline{inc}\right)+{\sum}_k{\hat{\delta}}_k{X}_{k1}+{\sum}_p{\hat{\varphi}}_p{\overline{Z}}_{p1} $$

Then it was calculated ${\hat{y}}_i^{1s}={y}_i-{\hat{y}}_i^x+y$, in which ${\hat{y}}_i^{1s}$ is the standardized demand for health care services, *y*_*i*_ is the observed demand for the use of the health care services of individual *i*, ${\hat{y}}_i^x$ is the expected demand given *X* and *y* It is the sample mean of real demand of health care services. After performing the standardization, ECI was calculated for both the current demand (*C*_*m*_) and predicted demand (*C*_*p*_) and HI was estimated as follows:

$$ HI=2{\int}_0^1\left[{L}_p(p)-{L}_m(p)\right] dp={C}_m-{C}_p $$

In which *L*_*p*_(*p*) is the CC for the predicted demand for health care services and *L*_*m*_(*p*) is the CC for the actual demand. The values of the IH range between − 2 and 2. When the HI is positive, it suggests that the inequality standardized by necessity shows inequities that favor the richest individuals.

Finally, econometric micro-decomposition methods were applied to determine the contribution of each factor to inequality in the use of health services, following the methodology developed by Van Doorslaer et al. [17, 18]. The decomposition was performed by ordinary least squares regression model, based on a linear approximation of the partial effects of each factor evaluated in the sample means. This approach allows us to identify which factors are associated with the pro-rich and pro-poor use of health care services and approximate their contribution to ECI.

## Results

Table 1 shows the main characteristics of the total population under study (*n* = 32,365). 52.6% of adults are female, between the ages of 35–49 years (26.6%) and half the population completed high school or a higher educational level (51.9%). Most adults have health coverage through social security (57.0%), and approximately three out of five adults are employed (62.7%). Nearly half reported having good health (42.9%), and just over half of them indicated having a low level of physical activity (54.7%). Half of the adults reported having used health care services (49.4%). Women, the elderly, and adults living in homes with 1 to 3 members report greater use of health care services compared to men, younger adults, and adults living in larger households, respectively. Likewise, adults covered by social security insurance and those who are unemployed reported greater use of health care services compared to those who are affiliated with other health insurance or are employed. Greater use of health care services was reported as income per capita increases, self-perception of health status is worse, or the number of chronic diseases increases. There are no differences in the use of health care services according to the size of the population grouping or according to geographical region.

**Table 1:** Descriptive statistics

	**Total**	**Reported using health care services**		
**Characteristics**	**n (%**^**a**^**)**	**n (%**^**b**^**)**	**C.I.95%**	**p value**
General population	32365 (100.00)	16226 (49.37)	48.30 - 50.43	
**Predisposing factors**				
Sex				
Male	14317 (47.43)	5914 (40.64)	39.07 - 42.20	<0.001
Female	18048 (52.56)	10312 (57.24)	55.84 - 58.65	
Age (in years)				
18-24	4341 (16.55)	1690 (40.17)	37.28 - 43.06	<0.001
25-34	7028 (21.90)	3155 (42.35)	40.09 - 44.61	
35-49	9013 (26.58)	4063 (45.45)	43.43 - 47.47	
50-64	6607 (19.82)	3707 (57.56)	55.29 - 59.83	
65 or more	5376 (15.15)	3611 (65.72)	63.24 - 68.20	
Married or cohabitating?				
Yes	17281 (58.13)	8579 (49.84)	48.44 - 51.25	0.299
No	15084 (41.87)	7647 (48.70)	47.07 - 50.33	
Educational level				
Up to incomplete primary	3561 (9.86)	1909 (50.96)	47.63 - 54.30	0.002
Complete primary and incomplete secondary	12287 (38.27)	5827 (45.66)	43.90 - 47.42	
Complete secondary or more	16517 (51.87)	8490 (51.80)	50.34 - 53.26	
Household size				
1-3 members	19124 (49.93)	10351 (54.77)	53.38 - 56.16	<0.001
4-6 members	11507 (42.41)	5197 (45.09)	43.36 - 46.83	
7 members or more	1734 (7.66)	678 (37.83)	33.87 - 41.79	
**Enabling factors**				
Type of health coverage^c^				
Social security insurance	19294 (56.96)	10685 (54.55)	53.16 - 55.95	<0.001
Private insurance	3622 (13.92)	1958 (53.60)	50.76 - 56.45	
Public insurance	9147 (29.12)	3454 (37.45)	35.48 - 39.43	
Currently employed?				
Yes	20060 (62.69)	9203 (44.91)	43.57 - 46.25	<0.001
No	12305 (37.31)	7023 (56.86)	55.14 - 58.58	
Urban population				
More than 1.5 million inhabitants	2862 (37.33)	1513 (50.23)	48.02 - 52.44	0.091
Between 500,001 y 1.5 million inhabitants	6093 (19.08)	3171 (50.53)	49.04 - 52.02	
Between 100,001 y 500,000 inhabitants	11220 (16.76)	5625 (48.50)	47.20 - 49.79	
Between 5,000 y 100,000 inhabitants	12190 (26.83)	5917 (47.88)	45.73 - 50.03	
Geographical region				
Greater Buenos Aires	2862 (37.33)	1513 (50.23)	48.02 - 52.44	0.101
Pampeana	9618 (33.43)	5000 (50.18)	48.33 - 52.03	
Northwest	6584 (10.24)	3051 (45.29)	43.77 - 46.81	
Northeast	4014 (7.34)	1973 (46.10)	43.98 - 48.22	
Cuyo	3339 (6.44)	1686 (50.15)	47.90 - 52.39	
Patagonica	5948 (5.22)	3003 (49.62)	47.97 - 51.27	
**Need factors**				
Self-perceived health status				
Excellent	3658 (12.08)	1396 (38.26)	35.30 - 41.22	<0.001
Very good	7477 (23.81)	3282 (44.16)	41.97 - 46.34	
Good	13967 (42.86)	6643 (46.58)	44.94 - 48.22	
Regular	6345 (18.11)	4172 (64.55)	62.21 - 66.90	
Bad	918 (3.12)	733 (82.12)	77.44 - 86.80	
Problems with mobility?				
Yes	3752 (11.00)	2713 (72.47)	69.64 -75.31	<0.001
No	28613 (89.00)	13513 (46.51)	45.38 - 47.65	
Feeling lonely or depressed?				
Yes	16616 (52.58)	8360 (49.54)	48.04 - 51.04	0.264
No	15166 (47.42)	7429 (48.31)	46.76 - 49.86	
Level of physical activity				
Intense	4522 (13.79)	2011 (42.77)	40.06 - 45.48	<0.001
Moderate	10107 (31.48)	4969 (49.73)	47.85 - 51.61	
Low	17467 (54.73)	9126 (51.00)	49.52 - 52.48	
Pain or physical discomfort?				
Yes	7934 (24.06)	5206 (65.64)	63.68 - 67.61	<0.001
No	24431 (75.94)	11020 (44.21)	42.98 - 45.44	
Suffers from high blood pressure?^d^				
Yes	10275 (34.33)	6318 (60.56)	58.71 - 62.41	<0.001
No	18291 (65.67)	8752 (47.42)	46.02 - 48.82	
Suffers from diabetes mellitus?^e^				
Yes	3347 (9.80)	2253 (68.52)	65.49 - 71.56	<0.001
No	28885 (90.20)	13941 (47.39)	46.26 - 48.51	
Suffers from chronic obstructive pulmonary disease?^f^				
Yes	1408 (4.34)	899 (62.80)	57.72 - 67.88	<0.001
No	30904 (95.66)	15302 (48.80)	47.71 - 49.89	
Suffers from chronic kidney disease?^g^				
Yes	1782 (4.76)	1149 (65.19)	60.85 - 69.52	<0.001
No	30520 (95.24)	15039 (48.55)	47.46 - 49.65	
Numbers of chronic diseases suffered				
None	19332 (61.67)	8312 (42.81)	41.44 - 44.18	<0.001
One	9758 (29.03)	5586 (55.84)	53.92 - 57.77	
Two	2814 (7.91)	1983 (72.08)	68.87 - 75.28	
Three or more	461 (0.13)	345 (75.82)	67.71 - 83.93	

Source: National Survey of Risk Factors (ENFR) 2013

^a^Column weighted proportion based on the expansion factor of the ENFR 2013

^b^Raw weighted proportion based on the expansion factor of the ENFR 2013

^c^n=32063; ^d^n=28566; ^e^n=32232; ^f^n=32312; ^g^n=32302

**Fig. 2:**
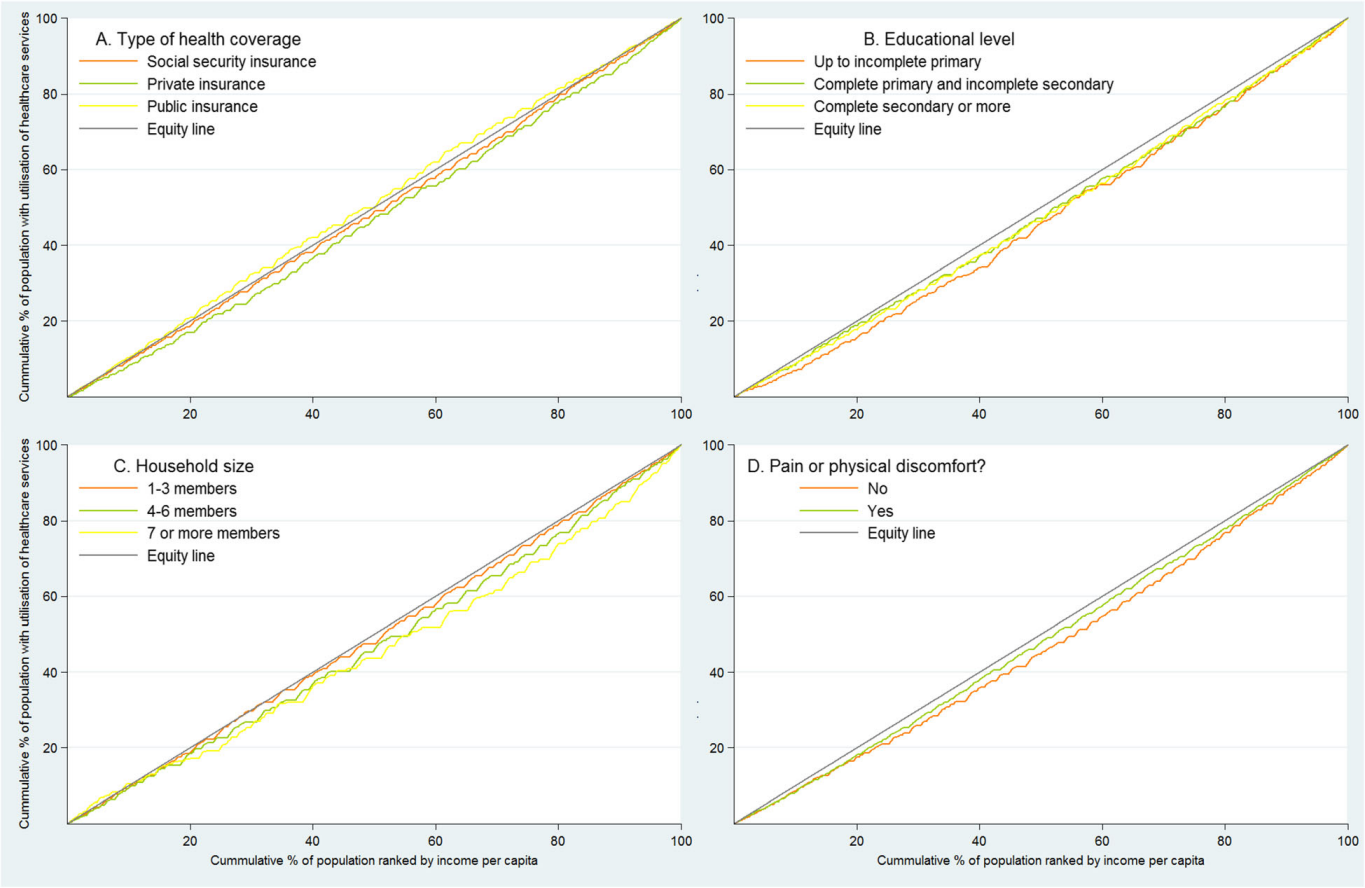
Concentration curves for the use of health care services in Argentina

Figure 2 shows the CC for the use of health care services estimated for some selected factors: type of health coverage, educational level, household size, and presence of pain or physical discomfort. In most of CC, a pro-rich inequality was identified, except for having public health insurance. In the subgroup of adults with private insurance, adults with incomplete primary education, and adults living in households with 7 or more members, the inequality tends to be more significant compared to adults with other types of insurance, adults with more education or adults who live in smaller households, respectively.

Table 2 describes the distribution, and inequality of the actual use, expected use according to need and standardized use according to the need for health care services. The actual use of health care services increases according to income quintile. The wealthiest quintile has a utilization of approximately 15 percentage points higher than the most impoverished quintile. The positive value of ECI (0.1223) indicates pro-rich inequality in the use of health care services. When utilization is adjusted by predisposing and enabling factors, the expected use of health care services is higher among people in poor quintiles. The difference in use between expected and actual use reflects an underutilization of the health care services by the individuals in poor quintiles. After standardizing the use of health care services for health needs, a positive HI was obtained (0.1296), indicating that even when individuals have the same needs, the use of standardized health care services as needed is higher among individuals of richer quintiles.

**Table 2:** Distribution and inequality of observed utilisation, expected utilisation based on need, and need-standardized utilisation of healthcare services

**Income per capita quintile**	**Observed utilisation**	**Expected utilisation based on need**	**Difference**	**Need-standardized utilisation**
I (poorest)	0.4192	0.5340	-0.1149	0.4290
II	0.4697	0.5141	-0.0444	0.4655
III	0.5038	0.5150	-0.0113	0.5019
IV	0.5448	0.5088	0.0361	0.5422
V (richest)	0.5659	0.4854	0.0806	0.5928
Average	0.4937	0.5131	-0.0194	0.5004
ECI / HI	0.1223			0.1296

*ECI* Erreygers concentration index; *HI* Horizontal inequity index

Table 3 shows the decomposition of the ECI, that is, the inequality in the use of health care services by need and non-need factors. Non-need factors contribute mostly to “pro-rich” inequality (77.84% of total pro-rich inequality), while need factors contribute negatively to pro-rich inequality (− 5.92% of pro-rich inequality). In particular, the most significant individual contribution to the pro-rich inequality in the use of health care services are the affiliation to social security insurance (30.66% of total pro-rich inequality), education (33.25%) and the per capita income (10.42% for the highest quintile) (for further details see Figure S1 in the Supplementary material).

**Table 3:** Decomposition of the Erreygers concentration index (ECI)

	*Contribution to the ECI for the utilisation of healthcare services*	
	*Contribution*	*Contribution as percentage*
**Need factors**		
Sex	-0.0029	-2.39%
Age 25-34 years	0.0004	0.32%
Age 35-49 years	0.0006	0.49%
Age 50-64 years	0.0011	0.89%
Age 65 years or older	0.0014	1.11%
Self-perceived health status: very good	0.0027	2.24%
Self-perceived health status: good	-0.0019	-1.52%
Self-perceived health status: regular	-0.0076	-6.25%
Self-perceived health status: bad	-0.0004	-0.29%
Problems with mobility	-0.0002	-0.16%
Hypertension	-0.0005	-0.39%
Diabetes mellitus	0.0001	0.11%
Chronic obstructive pulmonary disease	0.0000	-0.01%
Chronic kidney disease	-0.0001	-0.08%
Subtotal	-0.0072	-5.92%
**Non-need factors**		
Married or cohabiting	0.0001	0.07%
Household size: 4-6 members	0.0051	4.17%
Household size: 7 or more members	0.0011	0.89%
Complete primary and incomplete secondary	-0.0082	-6.71%
Complete secondary or more	0.0407	33.25%
Social security insurance	0.0375	30.66%
Private insurance	0.0054	4.38%
Currently employed	-0.0035	-2.89%
II income per capita quintile	0.0012	0.97%
III income per capita quintile	0.0000	0.01%
IV income per capita quintile	0.0027	2.20%
V income per capita quintile	0.0128	10.42%
Pampeana region	-0.0002	-0.17%
Northwest region	0.0005	0.43%
Northeast region	0.0002	0.16%
Cuyo region	-0.0001	-0.04%
Patagonica region	0.0001	0.05%
Subtotal	0.0952	77.84%
Residual	0.0344	28.08%

Values <0 suggest "pro-poor" utilization and >0 suggest "pro-rich" utilization

## Discussion

This study measured socioeconomic inequality and horizontal inequity related to the use of health care services in Argentina. The results show inequalities in the use of health care services, with the detriment of the vulnerable population. When utilization of health care services was standardized according to health needs, pro-rich inequality was identified. The main non-needs factors that contribute to the pro-rich inequality were education, social security insurance, and income.

In particular, our results suggest that the fragmentation of the health care system into three subsectors in terms of financing and service delivery has implications for health care inequality. Among those who have health coverage through social security, there is a marked pro-rich inequality in the use of health care services. This inequality could be because heterogeneous *Obras Sociales,* in terms of attention scale, average contribution by affiliate, and benefit coverage, are part of Argentina’s social security. These aspects correlate positively with labor income and professions of affiliates [11]. Something similar seems to occur in the private health subsector. In the absence of a regulatory framework for private insurance, private medicine companies compete by offering different health coverage plans and access to providers with differentiated prices, generating a subsector segregated by payment capacity. In general, the population that access to private insurances (and in particular the special coverage plans offered within each private insurance) has the highest income. This fact not only generates disparities in access, use, and quality of services between individuals covered by different subsectors but also between individuals within each subsector [52, 53].

On the other hand, our results suggest a negligible contribution of the decentralization of the health care system (represented here through analysis at the geographic region level) to pro-rich inequality. In a literature review on the topic, we identified a local study that concludes that municipal management capacities are a good predictor of the use of health care services in low-income populations [44], and a regional study that suggests that decentralization in Chile, Colombia and Bolivia relates to an improvement in some equity indicators [54]. However, as already mentioned, we carried out our analysis at the regional level (and not at the provincial level because of sample size), so, likely, our approach does not account for the effect that decentralization could have on inequality at a smaller geographical scale (for example at the provincial or municipal level).

Another factor that contributes to pro-rich inequality is formal education. A possible explanation could be the existence of high educational segregation in Argentina. Even though access to free and public education is guaranteed to the entire population, in the last decades, an important process of selective migration of students of high socioeconomic levels towards private education institutions has been identified [55, 56]. The private education institutions of Argentina present characteristics and strategies of education (educational content, the extension of the school day, extra-curricular activities, among many others) that differ from what is offered by the public education institutions [57]. In addition, given the differences in the teaching strategy and the socioeconomic composition of the students, it is likely that those who study in private institutions have a higher endowment of human capital and social capital than those who study in public institutions [58]. This could translate into better employment and income opportunities for this subpopulation, increasing access and utilization of health services.

In general, our results of socioeconomic inequality and inequity related to the use of health services are superior to those reported in previous studies in Argentina [38–40]. Differences in the socio-economic characteristics of the individuals considered in each study could constitute a possible explanation. Two studies [39, 40] used data on the use of services by older adults, so that comparability with our results may be limited. Despite this, our results on the determinants that most contribute to pro-rich inequalities (health coverage, education, and income) are similar to those reported by other studies at the local [38–40] and regional level, such as Brazil [59–62], Chile [63, 64], Colombia [65, 66], Ecuador [67, 68] and Mexico [69].

The findings of this study are relevant for policy discussion at a local and regional level. First, it is necessary to strengthen and expand public health coverage programs that are specific to vulnerable population subgroups such as pregnant women, children, or poor individuals. This process should involve the Nation, provinces and municipalities, who should agree on prioritized population groups, healthcare lines, and articulate the use of available resources. In the last 15 years, the Argentine government has implemented the *Nacer / SUMAR* Program, a pay-for-performance program that operates within the federal system of the country, and provides health coverage to pregnant women and children. Program evaluations have shown favorable results [70, 71], and it is currently considered as a platform to reach the UHC at the local level [46]. Secondly, the design and implementation of cross-subsidy mechanisms between the health subsectors, mainly between the public and social security subsectors, is required in order to compensate for the socio-economic inequalities observed among population groups, improving coordination and integration between sectors [7]. Local experience in this direction is the *Sistema Único de Recupero* (SUR, acronym in Spanish) Program, which consists of providing financial coverage for high-cost diseases within the union social security subsector. The Superintendence of Health Services, which is the national regulatory entity of social security institutions, manages this program. An external evaluation of this program suggests that it seems to have contributed to greater equity among OSN with different financing capacities [72].

Third, the design and implementation of benefits packages at prioritized pathologies level are suggested, in order to guarantee a package of high-quality health services to the entire population regardless of the type of coverage and place of residence. Some countries in the region have made progress in these initiatives as a means to reduce health care inequalities. For example, in 2002, Chile established a right guarantee mechanism known as the *Plan de Aseguramiento Universal de Garantías Explícitos* (AUGE, acronym in Spanish). On that occasion, a set of 57 protocolized pathologies was selected, and it was assumed that all citizens have access to the same treatments regardless of whether they are provided by the public system or by the private one, with satisfactory results in terms of reducing health inequalities [73].

This study has limitations that need consideration. First, the survey used (ENFR 2013) does not collect information on additional variables that could affect the effective use of health care services, such as accessibility to services, the perception of the quality of services by individuals, among others. Second, the survey used does not report information on the use of health care services by individuals under 18, so that subpopulations of interest, potentially vulnerable such as children and adolescents, were not included in our study. Third, the use of cross-sectional data in our analysis prevents us from discussing our findings in terms of potential causal relationships. Fourth, the ENFR 2013 does not present detailed information on household members (particularly sex and age), which does not allow to perform the analysis with per capita income adjusted for equivalent adult. However, we replicated our analysis following the equivalent adult adjustment criteria proposed by the Organization for Economic Cooperation and Development (OECD) [74], and the conclusions of our study remained unchanged. Fifth, the survey only provides information about health conditions by self-report, which could bias our estimates. The latest version of the survey (ENFR 2018, recently available) has two types of health condition measurements (by biomarkers and by self-report) for a subsample of individuals. According to the data of this survey, the self-report implies an underestimation for some health conditions, for example, 4.5 percentage points (p.p.) for the case of overweight or obesity, 5.8 (p.p.) for hypertension, and 4.3 (p.p.) for diabetes [75]. Based on this, future studies become necessary to analyze the implications of these differences in the health condition on the measurement of health inequality at the local level. Sixth, the validity of the Andersen theoretical model used in this study could face challenge based on the characteristics of the local health system, the socio-economic aspects of the population, and its epidemiological profile. In this regard, countries such as Mexico have adapted Andersen’s theoretical model to apply it to their local context [76]. However, local studies have been identified that have used this theoretical framework to explain the use of health care services [44–46], without making adaptations.

In conclusion, the Argentine health system shows pro-rich inequality in the use of health care services. To keep the commitment of “not to leave anyone behind”, it is necessary to design strategies to improve articulation between the three coverage subsectors, and national, provincial and municipal governments. The results showed here could provide lessons for countries with similar contexts and challenges in public health.

## Supplementary information

**Additional file 1: Table S1.** Basic indicators. Argentina, Latin American and the Caribbean countries (average) and Organization for Economic Cooperation and Development (OECD) country members (average).

**Additional file 2: Figure S1.** Decomposition of the Erreygers concentration index (ECI).

## Data Availability

The data utilized in this study was from the Third National Survey of Risk Factors 2013 (ENFR 2013) designed and compiled by the National Institute of Statistics and Census (INDEC) and the Ministry of Health of Argentina. Data are available at: https://www.indec.gob.ar/indec/web/Institucional-Indec-BasesDeDatos-2
