# Supplementary material for: Need and inequality in the use of health care services in a fragmented and decentralized health system: evidence for Argentina
Source: Int J Equity Health. 2020 Jul 31;19:67. doi: 10.1186/s12939-020-01168-6 (PMC7394688; doi:10.1186/s12939-020-01168-6)
Supplement: Supplementary file 1 — Additional file 1: Table S1. Basic indicators. Argentina, Latin American and the Caribbean countries (average) and Organization for Economic Cooperation and Development (OECD) country members (average). [file 12939_2020_1168_MOESM1_ESM.docx]

**Figure S1. Decomposition of the Erreygers concentration index (ECI)**
