# Supplementary material for: Need and inequality in the use of health care services in a fragmented and decentralized health system: evidence for Argentina
Source: Int J Equity Health. 2020 Jul 31;19:67. doi: 10.1186/s12939-020-01168-6 (PMC7394688; doi:10.1186/s12939-020-01168-6)
Supplement: Supplementary file 2 — Additional file 2: Figure S1. Decomposition of the Erreygers concentration index (ECI). [file 12939_2020_1168_MOESM2_ESM.docx]

| **Table S1. Basic indicators. Argentina, Latin American and the Caribbean countries (average) and Organization for Economic Cooperation and Development (OECD) country members (average)** | | | |
| --- | --- | --- | --- |
| **Indicator** | **ARG** | **LAC** | **OECD** |
| GDP per capita, PPP (international dollars $)^c^ | 18932.17 | 14473.53 | 39589.21 |
| Health expenditure as a percentage of GDP^a^ | 6.83 | 7.39 | 12.44 |
| Per capita health expenditure, PPP (international dollars $)^a^ | 1389.84 | 1080.88 | 4880.16 |
| Government health expenditure, as a percentage of GDP^a^ | 4.88 | 3.83 | 7.79 |
| Per capita government health expenditure, PPP international dollars $^a^ | 992.58 | 556.54 | 3058.65 |
| Private health expenditure as a percentage of total health expenditure^a^ | 28.01 | 47.47 | 39.01 |
| Per capita health expenditure, PPP international dollars $^a^ | 389.24 | 513.77 | 1904.14 |
| Out-of-pocket payments as a percentage of total health expenditure^a^ | 17.63 | 31.26 | 13.76 |
| Per capita out-of-pocket payment, PPP international dollars $^a^ | 245.00 | 333.99 | 702.92 |
| Universal health coverage index^a^ | 76.00 | 74.36 | 79.80 |
| Life expectancy at birth, total (years)^b^ | 76.58 | 75.44 | 80.11 |
| Population 0-14 years as a percentage of total population^c^ | 24.89 | 25.11 | 17.94 |
| Population 65 years or older as a percentage of total population^c^ | 11.20 | 7.88 | 16.81 |
| Neonatal mortality rate (per 1,000 live births)^c^ | 5.90 | 9.74 | 3.70 |
| Infantile mortality rate (per 1,000 live births)^c^ | 9.20 | 15.06 | 5.70 |
| Under 5 years mortality rate (per 1,000 live births)^c^ | 10.40 | 17.81 | 6.68 |
| Mortality from cardiovascular diseases, cancer, diabetes or chronic respiratory diseases between the ages of 30 and 70 (%)^b^ | 15.80 | 16.01 | 12.44 |
| Source: World Development Indicators, The World Bank |  |  |  |
| *GDP*: Gross Domestic Product; *PPP*: 2011 Purchasing Power Parity; *ARG*: Argentina; *LAC*: average of the Latin American and the Caribbean countries; *OECD*: average of the Organization for Economic Cooperation and Development country members. | | | |
| Values corresponding to the following years: ^a^2015, ^b^2016, ^c^2017 |  |  |  |
